# Osteonecrosis of the Jaw Associated with Obinutuzumab in a Patient with Preceding Follicular Non-Hodgkin’s Lymphoma

**DOI:** 10.3390/jpm15040138

**Published:** 2025-04-01

**Authors:** Katharina Theresa Obermeier, Thomas Frank, Tim Hildebrandt, Sven Otto, Philipp Poxleitner, Ina Dewenter

**Affiliations:** Department of Oral and Maxillofacial Surgery and Facial Plastic Surgery, Ludwig-Maximilians-University (LMU), Lindwurmstrasse 2a, 80337 Munich, Germany; katharina.obermeier@med.uni-muenchen.de (K.T.O.); thomas.frank2301@gmail.com (T.F.); tim.hildebrandt@med.uni-muenchen.de (T.H.); sven.otto@med.uni-muenchen.de (S.O.); philipp.poxleitner@med.uni-muechen.de (P.P.)

**Keywords:** immunotherapy, osteonecrosis, jaw, bone remodeling

## Abstract

**Background:** Obinutuzumab is a glycoengineered type II anti-CD-20 monoclonal antibody, which can be applied as immunotherapy in patients with follicular lymphoma. To our knowledge, this is the first reported case in the literature describing osteonecrosis of the jaw associated with CD20 monoclonal antibody therapy. **Methods:** The following case report describes a 39-year-old female patient under maintaining therapy with Obinutuzumab developing osteonecrosis of the jaw after tooth extraction. The necrotic area was located in the right mandible and was rated as a stage II osteonecrosis. **Results:** This case report should draw attention to the importance of dental follow-ups during aftercare of patients with Non-Hodgkin’s Lymphoma as well as to the relevant precautions for performing tooth extractions in such patients. **Conclusions:** As Obinutuzumab seems to be a contributing factor in the development of MRONJ, special attention has to be drawn to tooth extractions in such patients, which should only be performed with perioperative antibiosis, the least amount of trauma possible, always including the smoothening of sharp residual bone segments and a saliva-proof wound closure, as well as constant dental follow-ups.

## 1. Introduction

Medication-related osteonecrosis of the jaw (MRONJ) per definition is a condition of exposed or non-exposed necrotic lesions in the jaw over the course of 8 weeks as a result of treatment with antiresorptive or anti-angiogenic drugs without relation to radiotherapy. Such therapeutic agents are often used for prevention of bone metastases caused by tumor as well as osteoporosis therapy and tumor remission control [1,2,3,4]. There are five reported cases in the literature where chemotherapy itself is seen as a cause for what is also classified as MRONJ [5,6]. These cases simultaneously received corticosteroids or BP as well as Obinutuzumab. In our case, Obinutuzumab was the sole medication for more than 2 years [7]. Obinutuzumab itself is a glycoengineered type II anti-CD-20 monoclonal antibody, which can be applied as immunotherapy in patients with follicular lymphoma [8,9]. It is the successor of Rituximab, also a CD-20 monoclonal antibody, with a greater direct B-Cell inhibiting effect [10]. Follicular lymphoma is the second most common lymphoma in Western Europe. In general, therapy is complex and depends on the molecular characteristics; different chemotherapy regimens exist and can be adapted to the tumor stadium. CD20 surface antigens on B-cell lymphomas are targeted by Rituximab, which plays an important role in therapy and overall survival [11]. Advanced tumor stages are treated with Rituximab in combination with chemotherapy. One of the most applied chemotherapy regimens is R-CHOP (Rituximab, Cyclophosphamid, Hydroxydaunorubicin, Vincristin, Prednisolon). Recently, clinical studies evaluating CAR (chimeric antigen receptor) T-cell therapy targeting CD19/CD20 have proven to be highly successful as well [12]. Obinutuzumab was developed to potentiate activity and overcome resistance. Marcus et al. 2017 [8] showed, in a large collaborative trial, that the progression-free survival with Obinutuzumab compared to Rituximab was significantly higher [8]. However, side effects occurred more often with Obinutuzumab. The incidence of infusion-related reactions has doubled compared to the use of Rituximab [10]. Effects on the bone remodeling by CD-20 monoclonal antibodies have been described in the literature. The development of jaw necrosis by these bone-modeling phenomena has not yet been described.

## 2. Case Report

**Figure 1:**
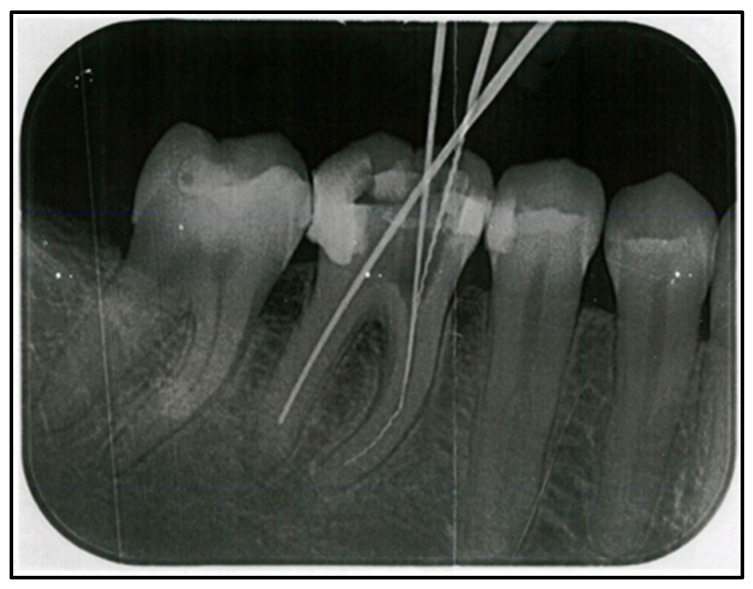
Pre-operative periapical radiograph of tooth 46 with three files taken at the dental practice during root canal treatment.

A 39-year old female patient presented herself in the Department of Oral and Maxillofacial Surgery of the Ludwig-Maximilians-University Munich with pain in the region of tooth 46, which had previously been root-canal-treated by the general dental practitioner (Figure 1).

For her medical history, she reported Hepatitis B Virus (HBV) constantly under the detection limit due to ongoing therapy with Entecavir. Furthermore, the patient underwent chemotherapy following the CHOP-Scheme combined with Obinutuzumab from April to August of 2018 due to a follicular Non-Hodgkin’s Lymphoma Grade 3a. CHOP consists of Cyclophosphamide, Hydroxydaunorubicine, Vincristin, and Prednisone. Additionally, she received maintaining therapy with Obinutuzumab 1000 mg i.v. every two months via a central venous port. Complete remission was achieved under the described therapy. The jaw bone was not affected during diagnosis and follow-up. Concomitant medication consisted of 50 mg of levothyroxine as well as 960 mg of cotrimoxazole. The patient did not present with osteoporosis or premature menopause and never received any antiresorptive medication. Moreover, the patient suffered from polyneuropathy of both legs. In respect to these secondary diagnoses, the patient was hospitalized in day-care clinic but did not receive any further specific drug treatment. In February 2023, the tooth 46 appeared clinically infected, hence indicating an extraction in the day-care setting making use of modeling osteotomy and gingivoplasty. Extraction in local anesthesia was not possible because of a local anesthetics allergy; therefore, it was performed under sedation. Aftercare procedures included the prescription of Amoxicillin and Clavulanic acid 875/125 mg 1-0-1 for five days as well as a maximum of 600 mg of Ibuprofen three times a day in case of pain. Intra- and postoperatively, there were no hemorrhagic complications with respect to the fact of the patient pre-operatively suffering from mild thrombocytopenia of 130.000–140.000/µL. She was therefore discharged from the hospital on the same day.

**Figure 2:**
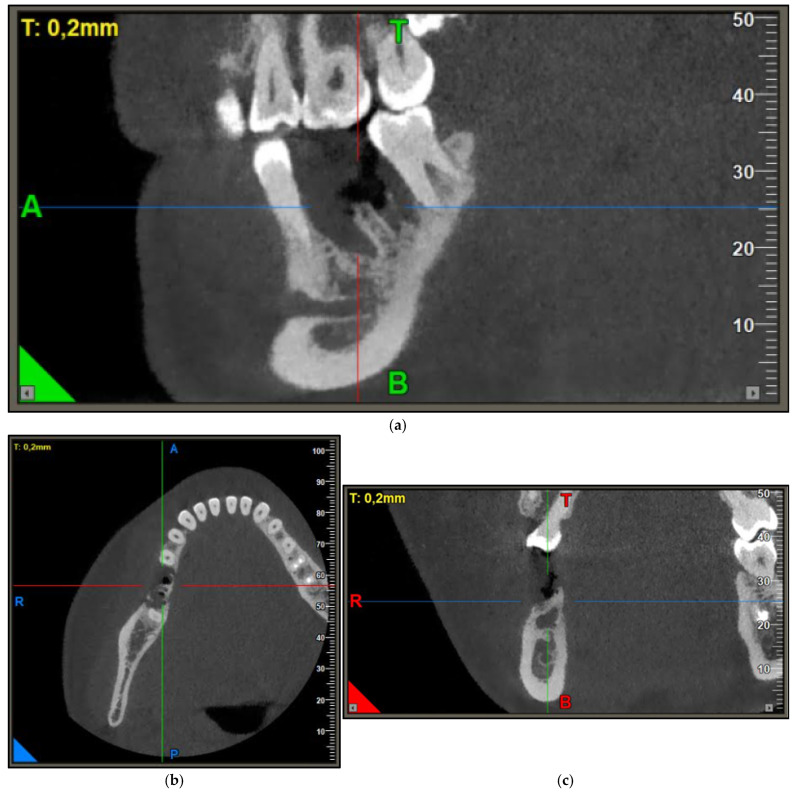
Pre-operative CBCT of necrotic site: (**a**) Extraction socket of tooth 46, devitalized bone region 45–47, sagittal view of the situation. (**b**) Axial view. (**c**) Coronal view.

**Figure 3:**
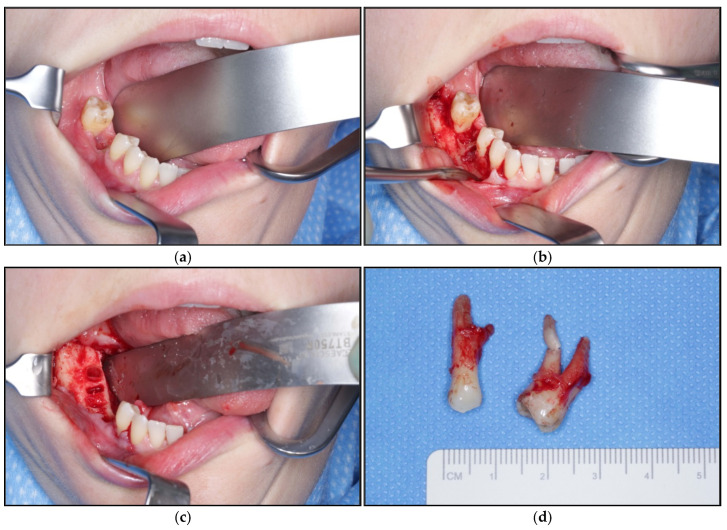
Intra-operative clinical photographs: (**a**) Before preparation of flap. (**b**) Situation before surgical debridement. (**c**) Situation after surgical debridement and modeling osteotomy. (**d**) Extracted teeth 45 and 47.

In the further course, the patient again reported pain in the region of the osteotomy during checkup at our department six weeks after tooth extraction. Clinically, there was discrete erythema of the gingiva with an area of exposed bone vestibular to tooth 46. After clinical examination, a dental cone beam CT was performed due to the suspected diagnosis of a MRONJ Stage II associated with Obinutuzumab (CBCT Figure 2). The CBCT showed devitalized bone in the region 45 to 47 with perifocal inflammation. Pictures of the intraoral findings were taken prior to planning of the surgery (Figure 3a). Bone turnover markers were not assessed.

## 3. Treatment

Surgical resection of the necrosis and modeling osteotomy under general anesthesia was performed seven weeks after the tooth extraction of tooth 46 (Figure 3). One bone fragment of 0.9 × 0.3 × 0.3 cm^3^ and one piece of fragmented gray tissue with a connected diameter of 0.9 cm were taken and sent to the pathologic institute for examination in order to rule out a potentially underlying malignant process, followed by microbiologic sampling. Intra-operatively, teeth 45 and 47 were discovered to have lost bony attachment on the vestibular side as a result of osteonecrosis in the whole area in addition to visible granulation tissue. Furthermore, both teeth showed stage II mobility. Therefore, 45 and 47 were extracted following modeling osteotomy, including the removal of granulation tissue. Removal of the necrotic bone was performed, without infringement of the bone’s continuity, with bleeding, and vital bone becoming visible. Sharp bone contours were removed. The consecutive local plastic coverage of the area was achieved by preparation of the aforementioned mucoperiosteal flap, affixed with a braided absorbable 3.0 suture and the use of a continuous and a mattress suture also with the same material. With these measures, a three-layer, tension-free, and saliva proof wound closure was achieved.

Surgery was performed under perioperative antibiotic prophylaxis (Sultamicillin) 2000/1000 mg intravenously, and the patient could be discharged from hospital 4 days later in a stable condition with wounds free of irritation.

## 4. Outcome and Follow-Up

The results of the pathologic examination revealed that the gray tissue sample consisted of granulation tissue with a high amount of capillaries and infiltration with lymphoplasmacellular inflammation as well as deeper layers of stroma with infiltrates of granulocytic inflammation.

The decalcified bone fragment confirmed the clinical diagnosis of MRONJ, revealing a non-vital, compact bone with glandular-like depletions of bacteria, partly also showing some form of granulation tissue in the deeper medullary spaces.

There were no signs of malignity or epithelial dysplasia.

An analysis of the microbiological sample was performed and showed no anaerobic bacterial growth but only aerobic oral flora.

During a short-term follow up of six months, no remission occurred in terms of wound dehiscence or necrosis. Following consultation with colleagues in oncology, it was decided to resume the administration of Obinutuzumab for the maintenance treatment of follicular lymphoma.

## 5. Discussion

Bisphosphonates, such as alendronate and zoledronic acid, have been extensively investigated in the context of medication-related osteonecrosis of the jaw (MRONJ). The reported incidence of MRONJ in patients receiving bisphosphonate therapy ranges from approximately 1% to 15%. Patients receiving denosumab for osteoporosis generally exhibit a lower reported incidence of MRONJ, with rates ranging from less than 0.1% to 5% in various studies. Patients receiving denosumab in higher doses due to bone metastases generally present with a higher risk of MRONJ.

To our knowledge, MRONJ as a side effect of treatment with Obinutuzumab has not been reported yet in the literature. For the right treatment decision, it is essential to prove the diagnosis with a pathological examination. Therefore, suspected bone lesions should be evaluated by a panoramic radiograph as well as CBCT or CT if necessary [11]. Obinutuzumab is a third-generation monoclonal antibody that binds to the CD20 protein on the cell surface of B lymphocytes. In follicular lymphoma, B lymphocytes divide far too rapidly, thereby displacing healthy cells in the bone marrow [8]. Obinutuzumab supports the body’s own immune system to recognize and destroy the malignant cells [13]. Obinutuzumab can be classified as an immunomodulating drug often used in the chemotherapy of NHL and as a remission-controlling drug in follicular NHL patients. Even though to our knowledge MRONJ, as a side effect of Obinutuzumab treatment, has not been reported yet, there is evidence that anti-CD20-mediated B cell depletion is associated with bone preservation in lymphoma patients, therefore suggesting an influence on bone metabolism [14]. There has been evidence that rituximab, the predecessor of Obinutuzumab, reduces osteoclast activity, as assessed by serum markers of bone resorption [15]. Through the secretion of a receptor activator for a nuclear factor κ B ligand (RANKL) and osteoprotegerin (OPG) B, lymphocytes have the ability to regulate bone metabolism [16]. In addition, it has been shown that treatment with anti-CD20 antibodies was associated with preservation of bone mass in patients with follicular lymphoma who received rituximab maintenance [14]. Anti-CD20 treatment in a murine model resulted in decreased expression of RANKL in the whole bone and bone marrow of anti-mCD20-treated mice, as well as lower serum levels of RANKL [14]. In addition, the anti-angiogenic properties of maintenance therapy may also be important in the pathogenesis of osteonecrosis of the jaw. Recently, an increased risk of more severe infections has also been described with the administration of CD20 antibodies such as rituximab [17]. These described mechanisms resemble the pathogenesis of reduced bone metabolism in MRONJ patients that were treated with denosumab, therefore indicating that the impaired bone metabolism in Obinutuzumab-treated patients could be a risk factor for the development of MRONJ lesions.

When examining the pathogenesis of MRONJ lesions, several theories have been proposed, particularly in relation to the medications involved. In the case of bisphosphonates, it is postulated that a decrease in pH following infection, such as that which occurs after tooth extraction, results in the release and accumulation of bisphosphonates within the acidic environment. This process subsequently compromises bone metabolism, ultimately leading to the development of MRONJ [18]. Because of the drugs’ high affinity for tissues of low pH (Osteoclasts pH 1–2), they are expected to also show a high affinity to the infected bone.

By definition, drug-associated necrosis is an eight-week bone denudation with a history of known antiresorptive therapy and exclusion of tumor-therapeutic irradiation in the head and neck region; in our case, only seven weeks were given. On the other hand, the histopathologic findings are clear; avital bone and empty lacunae, consistent with a MRONJ, were described.

When observing treatment strategies, the management of necrotic areas in MRONJ cannot be effectively accomplished solely using antibiotics. Conservative treatment approaches for MRONJ demonstrate a limited success rate of only 14.9% in terms of mucosal healing [4]. In contrast, surgical therapy achieves a significantly higher success rate of 91.6%, with “successful” defined as the resolution of the disease [19]. This highlights the preference for a surgical approach, as undertaken in this case, to ensure the complete intraoperative resection of the necrotic bone. Additionally, the use of tetracycline autofluorescence (such as preoperative administration of doxycycline) combined with bone autofluorescence is considered a safe procedure for visualizing necrotic or infected areas, although it was not utilized in this particular case. However, it is frequently employed in our department [20].

The exact mechanism by which denosumab may contribute to the development of ONJ is not yet fully understood. By binding to RANKL, denosumab effectively inhibits the interaction between RANKL and its receptor, RANK, thereby suppressing osteoclast activation and impeding the differentiation of precursor cells into mature osteoclasts. Unlike BPs, denosumab does not accumulate in the bone tissue and has a relatively short half-life of approximately one month in the bloodstream. Although it has a shorter inhibitory effect on osteoclasts compared to BPs, it exhibits higher potency in inhibiting osteoclast activity.

In patients receiving therapy with denosumab who develop MRONJ, the same therapeutic regime is generally followed. Whether this treatment shows preferable effects also in MRONJ patients with Obinutuzumab—which might have similar effects on bone metabolism—still needs to be evaluated. The question arises whether the MRONJ lesion in our patient was just a form of a one-time incidence or if there really is a causality behind the drug intake and the appearance of osteonecrosis. It must be taken into account that osteonecrosis of the jaw can also appear more or less idiopathic after tooth extraction without a particular metabolic cause in the bone. This has already been seen and described by some authors [21,22]. In addition, Loncar et al. described cases of ONJ caused by materials used in root canal treatment for devitalization of the pulp, which were pressed throughout the apex of the root [22]. Furthermore, the formation of MRONJ lesions have also been described to be triggered by endodontic failure in oncologic patients, including root canal underfilling, root canal overfilling, root perforation, and root fracture.

In fact, the last intravenous dose of Obinutuzumab was given nearly two months before tooth extraction but only one month after root canal treatment at the patient’s dentist. The half-life of Obinutuzumab is known to be about 28 days [10]. This could even slightly indicate towards the root canal treatment being the inflammatory cause for the primary onset of the necrotic process instead of the tooth extraction. It also cannot be categorically ruled out that her COVID-19 infection, after which the patient tested negative soon before the tooth extraction, had an impact on the development of the MRONJ lesion as well. Apart from the osteonecrosis it has to be mentioned that it is possible for follicular lymphoma patients to obtain Obinutuzumab-related Acute Thrombocytopenia, which has already been described in the literature [23]. However, for our patient, it is more likely that her thrombocytopenia derives of HB Virus, because the number of thrombocytes has always been in a steady state since her medication with antiviral drugs was started, and the onset of thrombocytopenia in this case is not correlated in terms of time with the Obinutuzumab infusions.

To summarize, it is of utmost importance for patients receiving Obinutuzumab therapy to attend continuous dental follow-ups in order to restore dental foci as early and with as much caution as possible, as mechanisms of anti-CD 20 antibodies have already been described to impair bone metabolism. As Obinutuzumab seems to be a contributing factor in the development of MRONJ, special attention has to be paid to tooth extractions in such patients, which should only be performed with perioperative antibiosis, the least amount of trauma possible, always including the smoothening of sharp residual bone segments, and a saliva-proof wound closure, as well as constant dental follow-ups.

## Data Availability

Data is contained within the article.
